# A novel nano-iron supplement to safely combat iron deficiency and anaemia in young children: The IHAT-GUT double-blind, randomised, placebo-controlled trial protocol

**DOI:** 10.12688/gatesopenres.12866.2

**Published:** 2018-10-11

**Authors:** Dora I.A. Pereira, Nuredin I. Mohammed, Ogochukwu Ofordile, Famalang Camara, Bakary Baldeh, Thomas Mendy, Chilel Sanyang, Amadou T. Jallow, Ilias Hossain, James Wason, Andrew M. Prentice

**Affiliations:** 1 Department of Pathology, University of Cambridge, Cambridge, CB2 1QP, UK; 2 Medical Research Council Unit The Gambia at the London School of Hygiene & Tropical Medicine, Banjul, The Gambia; 3 MRC Biostatistics Unit, Institute of Public Health, University of Cambridge, Cambridge, CB2 0SR, UK; 4 Institute of Health and Society, Newcastle University, Newcastle, NE2 4BN, UK

**Keywords:** iron supplementation, children, IHAT, microbiome, iron deficiency, IDA, anaemia, diarrhoea

## Abstract

**Background: **Iron deficiency and its associated anaemia (IDA) are the leading forms of micronutrient malnutrition worldwide. Here we describe the rationale and design of the first clinical trial evaluating the efficacy and safety of an innovative nano iron supplement, iron hydroxide adipate tartrate (IHAT), for the treatment of IDA in young children (IHAT-GUT trial). Oral iron is often ineffective due to poor absorption and/or gastrointestinal adverse effects. IHAT is novel since it is effectively absorbed whilst remaining nanoparticulate in the gut, therefore should enable supplementation with fewer symptoms.

**Methods:** IHAT-GUT is a three-arm, double-blind, randomised, placebo-controlled phase II trial conducted in Gambian children 6-35 months of age. The intervention consists of a 12-week supplementation with either IHAT, ferrous sulphate (both at doses bioequivalent to 12.5 mg _Fe_/day) or placebo. The trial aims to include 705 children with IDA who will be randomly assigned (1:1:1) to each arm. The primary objectives are to test non-inferiority of IHAT in relation to ferrous sulphate at treating IDA, and to test superiority of IHAT in relation to ferrous sulphate and non-inferiority in relation to placebo in terms of diarrhoea incidence and prevalence. Secondary objectives are mechanistic assessments, to test whether IHAT reduces the burden of enteric pathogens, morbidity, and intestinal inflammation, and that it does not cause detrimental changes to the gut microbiome, particularly in relation to *Lactobacillaceae*, *Bifidobacteriaceae* and *Enterobacteriaceae*.

**Discussion: **This trial will test the hypothesis that supplementation with IHAT eliminates iron deficiency and improves haemoglobin levels without inducing gastrointestinal adverse effects. If shown to be the case, this would open the possibility for further testing and use of IHAT as a novel iron source for micronutrient intervention strategies in resource-poor countries, with the ultimate aim to help reduce the IDA global burden.

**Registration: **This trial is registered at clinicaltrials.gov ( NCT02941081).

## Introduction

Iron deficiency anaemia (IDA) remains the most common forms of micronutrient malnutrition in the world today. Globally, IDA is estimated to affect 1.24 billion people, the majority of whom are children and women from resource-poor countries, and is responsible for an estimated loss of 35 million disability-adjusted life years (DALYs) (1.5% of total DALYs) ^1– 3^. IDA is estimated to cause more years lived with disability (YLD) than all other micronutrient deficiencies, haemoglobinopathies and haemolytic anaemias combined, and is the leading contributor to YLD in most low-income countries ^1^. Most sub-Saharan Africa countries have an anaemia prevalence above 40% in young children and pregnant women, a severe public health problem according to the World Health Organization (WHO) ^4^. Iron deficiency is frequently exacerbated by concomitant parasitic and bacterial enteric infections and, together, these account for the majority of anaemia cases in developing countries ^5– 7^. The effects of anaemia on child cognition are also well recognised with several trials finding a combined 1.73 lower IQ points per 1 g/dL decrease in haemoglobin ^8^. Even mild iron deficiency in the absence of anaemia appears to impair intellectual development in young children and is lowering national IQs, whilst overt IDA is associated with increased risk of serious morbidity, poor motor and mental development in children and impaired immunity.

### The problem

Iron supplementation with simple ferrous salts is cheap and widely available, but constitutes a non-physiological approach to providing iron that has been associated with significant side-effects and adverse events ^9– 15^. Data and meta-analysis from trials involving nearly 10,000 young children, mainly from resource-poor countries, have consistently shown that conventional oral iron supplements used to treat IDA are associated with increased infection, including bloody diarrhoea ^10, 13, 16, 17^, detrimental changes to the gut microbiome and gut inflammation ^17– 20^. Therefore, in countries with poor infection control, iron supplementation in young children could further increase the burden from enteric infection and environmental enteropathy (i.e. persistent gut damage and inflammation that leads to malabsorption), which is a major cause of growth failure in children in resource-poor environments and may later exacerbate the risk of IDA ^21, 22^. Consequently, children living in areas where enteric infection is endemic remain without an effective and safe cure for ID and IDA, and, as such, it is perhaps not surprising that, despite considerable investment and effort, we have been unable to reduce the burden of this disease in young children in sub-Saharan Africa ^23^.

### Rationale and aims

Since 2005, we have been developing an engineered analogue of natural food iron as an alternative iron supplement. Iron hydroxide adipate tartrate or IHAT, is completely different from other iron compounds currently used in supplementation or home fortification strategies since it defies the established dogma that iron absorption requires ionic solubilised iron to be taken up by the duodenal enterocytes. The novelty of IHAT, is precisely that it is not a soluble compound nor does it require solubilisation in the stomach prior to uptake by the enterocytes as it is taken up as whole nanoparticles by endocytosis ^24, 25^, similarly to that proposed for dietary plant ferritin ^26– 28^. This means that the unabsorbed fraction of the compound that transits to the lower gut, which is usually at least 70% of all ingested oral iron, irrespective of the form, will remain nanoparticulate and, therefore not soluble, and as such should not be available to promote significant pathogen growth and tissue inflammation ^29, 30^. A crucial aspect of the IHAT nanostructure is that once it has entered the enterocyte it is sufficiently labile to break down effectively inside lysosomes/endosomes and deliver its iron because the native iron oxo-hydroxide structure (i.e. ferrihydrite) in IHAT has been purposely destabilised with the incorporation of dietary tartaric and adipic acids ^24, 25, 31^, much in the same way as what occurs in the ferritin iron core due to interactions with the amino-acid residues in the protein shell ^32^. Our pre-clinical and early-clinical data indicates that IHAT is effectively absorbed in humans, corrects IDA in animal models, is not redox reactive and does not have a detrimental impact on the gut microbiome ^24, 29– 31, 33^.

### Aims

The main purpose of the IHAT-GUT study is to determine whether supplementation with IHAT safely corrects IDA in young children compared to the present standard of care. We hypothesise that 12-weeks supplementation with IHAT will correct iron deficiency and improve haemoglobin levels in young children without causing diarrhoea or inducing intestinal inflammation and detrimental changes in the gut microbiome.

Here we summarise the protocol for the IHAT-GUT study, the first clinical study to assess the efficacy and safety of IHAT in anaemic children living in resource-poor rural areas of The Gambia.

## Protocol

### Hypothesis and objectives

We will test the hypothesis that supplementation with IHAT eliminates iron deficiency and improves haemoglobin (Hb) levels in young children without increasing diarrhoea or promoting negative changes in the gut microbiome or inducing gut inflammation.

The IHAT-GUT study has four primary combined objectives to test efficacy and safety of IHAT. The primary objectives in terms of efficacy for this trial are to test non-inferiority of IHAT in relation to ferrous sulphate at correcting ID and improving Hb levels after 12 weeks of supplementation. The primary objectives for safety are to test superiority of IHAT in relation to ferrous sulphate and non-inferiority in relation to placebo based on moderate-severe diarrhoea incidence and prevalence.

Secondary objectives of the IHAT-GUT trial are to test whether IHAT is non-detrimental with respect to enteric pathogen burden, does not increase morbidity, does not decrease abundances of *Lactobacillaceae* and *Bifidobacteriaceae* relative to *Enterobacteriaceae*, and does not cause intestinal inflammation.

### Study design and setting

***Design.*** The IHAT-GUT trial is a three-arm, parallel, randomised, placebo-controlled, double-blind study with iron supplementation in young children with mild to moderate iron deficient anaemia. Ferrous sulphate (FeSO _4_), the current standard of care for iron supplementation, is used as the active comparator at the conventional daily dose of 12.5mg iron ^34^.

Children are randomised (1:1:1) to IHAT, FeSO _4_ or placebo, and each arm includes an intervention period of 12 weeks. The daily iron dose is 12.5 mg elemental iron bioequivalent.

***Trial governance***. The trial is being conducted in accordance with the ethical principles that have their origin in the Declaration of Helsinki, and that are consistent with the International Conference on Harmonisation (ICH) requirements for Good Clinical Practice (GCP), and the applicable regulatory requirements. The study is sponsored by the London School of Hygiene and Tropical Medicine (LSHTM) and is conducted at the Medical Research Council (MRC) Unit The Gambia at LSHTM (MRCG).

***Setting***. The study population in IHAT-GUT are children under the age of 3 years living in the north bank rural communities in the Upper River Region (URR) of The Gambia in West Africa. The URR has an approximate population of 200,000, and only one major town, Basse, and is otherwise typical of rural sub-Saharan Africa. The temperature varies between 15°C and 40°C, but can reach as high as 46°C in the north bank. There are distinctive dry (November to May) and wet (June to October; mean annual rainfall = 876 mm) seasons. According to the most recent Gambia Demographic and Health Survey ^35^, the URR has the highest under-5 mortality rate in the country (92 deaths per 1000 livebirths), the highest percentage of severely malnourished children (7–11%), and the highest prevalence of malaria and anaemia in children under 5 years (4.5% and 82.5%, respectively). Severe anaemia in children is highly prevalent in these communities, with 22.4% of children under 5 years having haemoglobin below 8 g/dl ^35^. Diarrhoeal diseases are also common ^36, 37^. Therefore, there is a clear clinical need for safe and effective iron supplementation strategies in these communities.

**Figure 1.**
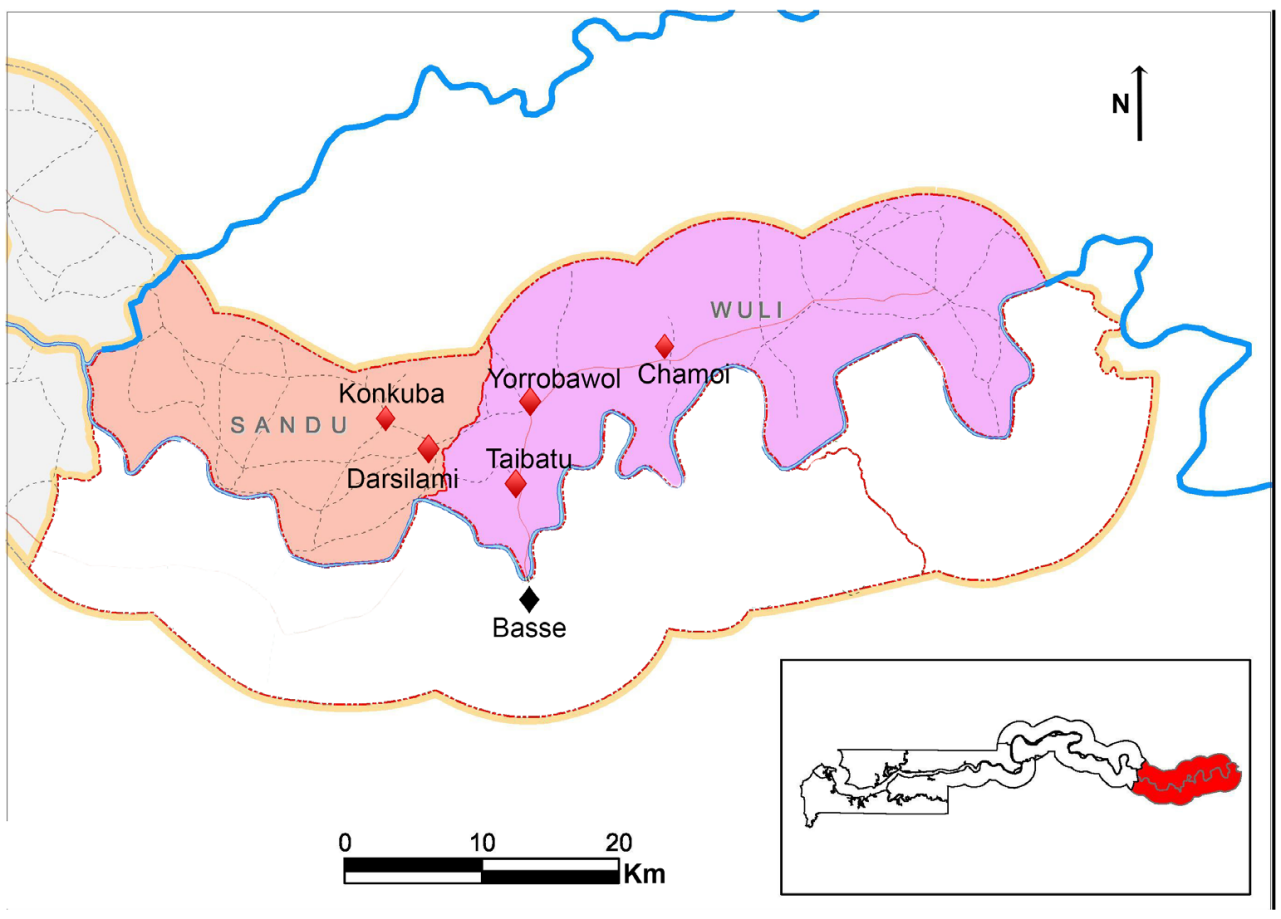
Map of Upper River Region in The Gambia, with location of study clinical facilities. Study samples are collected at one of the clinical facilities, Yorrobawol health center, Darsilami community health post, Konkuba community health post, Taibatu health post and Chamoi Health Center, and transported to the study laboratory in Basse for sample processing and analysis, and from there to other laboratories for further analysis. Map adapted from the Atlas of the Gambia 2004.

The study area includes 45 villages in the Wuli and Sandu districts ( Figure 1), situated on the north bank of the river Gambia, approximately 400 km east of the capital Banjul, with a population of approximately 2800 children aged 6–35 months. All communities have access to borehole tap water at central places. Study specimen samples are collected at one of the study health clinics: Yorrobawol health center, Darsilami community health post, Konkuba community health post, Taibatu health post and Chamoi Health Center ( Figure 1). Samples are transported in cold boxes to the study laboratory in Basse for sample processing and analysis, and from there to the Keneba and Fajara laboratories, or to external laboratories for further analysis.

### Study participants

Participants are young children and the study protocol is explained to their parents orally by the field staff. An impartial witness is present during consent for all illiterate parents and also signs the consent form next to the parent’s thumb print. No children start any study specific procedure without full, written informed consent being first obtained. A copy of the study information sheet and signed consent form is provided to those consented ( Supplementary File 1). Parents are free to withdraw their consent at any time. Participants must meet all of the inclusion criteria and none of the exclusion criteria to be eligible for the trial.

*Inclusion criteria*: Apparently healthy; 6–35 months old; free of malaria; with IDA defined as 7≤ Hb <11 g/dl and ferritin <30 μg/l ^38^; resident in the study area (and planning to remain in the study area for the duration of the trial); able and willing to comply with the study protocol; informed consent given by parent.

*Exclusion criteria*: Congenital anomalies/birth defects (except minor external congenital malformation); severe malnutrition (z-scores for length/height-for-age (HAZ), weight-for-age (WAZ), weight-for-length/height (WHZ) <-3 standard deviations (SD); shock syndrome; chronic conditions; sickle cell and thalassaemia; currently participating in another study; currently taking iron supplements/multiple micronutrient supplements; currently experiencing moderate-severe diarrhoea, defined as those diarrhoea episodes where (i) the child passes more than five loose or watery stools per day, (ii) there is blood in the stool (dysentery), or (iii) the child shows signs of clinical dehydration (assessed by the study nurse based on physical signs such as little or no urination, sunken eyes, and skin that lacks its normal elasticity).

### Recruitment and screening

Prospective participants are identified through the enumeration data collected by the study field team in the 45 study villages. The communities and regional health teams in the study area have been sensitised to the study. The field team visits the parents of all young children identified as prospective participants to explain the study and answer any questions they may have, those interested in taking part in the study are then invited to attend a screening visit at one of the study health facilities. At screening, the child is physically examined by a study nurse or clinician; those potentially eligible to take part in the study have their height and weight measured and a finger prick blood sample collected for Hb measurement and rapid diagnostic testing (RDT) for malaria. Children with Hb <7 g/dl or those severely malnourished cannot be enrolled and are referred to the regional health centre for treatment according to national guidelines. Malaria-positive children (positive RDT and confirmation by blood film) are not enrolled and will be treated according to national guidelines. If HAZ, WAZ, WHZ are all above -3 SD, 7≤ Hb <11 g/dl and the RDT is negative, then a small venous blood sample is collected by the study nurse to send to the laboratory for confirmation of Hb levels and determination of serum ferritin. If 7≤ Hb <11 g/dl and serum ferritin < 30 ng/ml, the child is invited to a pre-enrolment day back at the clinic (Day 0), which is 4–5 weeks after the screening visit, for a finger prick to confirm absence of malaria by RDT and haemoglobin concentration.

### Randomisation and blinding

Randomisation is based on a stratified block design to achieve group balance in terms of age and baseline haemoglobin concentration. Based on the assessments at the pre-enrolment day (Day 0), each child is categorised into two Hb classes (below or equal to/above the median Hb for the recruited cohort) and also according to age into 3 classes (6–11 months, 12–23 months and 24–35 months). This divides children into 6 different strata and in each strata the children are randomly assigned to one of the three study treatment arms (1:1:1 ratio) using R version 3.4.3 and a block randomisation approach with fixed block size determined by age and Hb levels.

Randomisation is performed by the study statistician who remains blinded to the treatment codes. Participants, parents and the entire study team are blinded as to which intervention/treatment arm participants belong to. Each child has a unique intervention code which is also their study ID/randomisation number, this means that the study team is unaware of which children belong to the same treatment arm. The child study ID is the same code that is used to label the capsules for the study treatment they receive (see below). If emergency unblinding is required, only the particular study subject in question will be unblinded, since each participant has a unique treatment code. Following investigation by the Data and Safety Monitoring Board (DSMB) of an emergency unblinding case, and only at the request of the DSMB, we may also unblind a whole treatment arm or the entire study. In all cases, someone independent from the trial team will perform the unblinding.

### Interventions

In each arm, children receive a daily dose of either IHAT, FeSO _4 _or placebo for 12 weeks. Children in the ferrous sulphate arm receive 12.5 mg elemental iron equivalent daily, as FeSO _4_.H _2_O, in line with WHO recommendations ^34^ and Gambian national guidelines for the 6–35 months age group. Children in the IHAT arm receive 20 mg elemental iron equivalent daily, which is the bioequivalent dose considering the bioavailability of IHAT relative to FeSO _4_. Children in the placebo group receive ca. 30 mg pharmaceutical grade sucrose daily. All three materials are formulated with 6 mg of a food colourant for colour matching and each daily dose is contained in a powder-filled easy-open capsule. Capsules for each treatment are packed in medicine bottles, each bottle containing enough capsules for one child for the entire study duration, and these bottles are individually labelled with the child’s unique study ID, which is their randomisation code, as described above. All capsules appear identical and the powders inside them do not have any apparent visual differences in texture or colour. Every day the field worker visits each child and provides them with the daily dose of their allocated treatment. For each child, the field worker opens one of their allocated capsules and adds all its contents to a small amount of a local juice drink, in a disposable plastic cup, immediately before administration directly into the child’s mouth. There are no apparent differences in the colour, turbidity or taste of the powder and juice mixes. Before administration, the mother is encouraged to feed the child so that the supplements are not ingested on an empty stomach.

Besides the study interventions mentioned here, children do not receive any other concomitant interventions or supplements during the 12-week intervention period, but all children are followed up weekly by the study clinical team, and any clinical conditions are treated following national guidelines.

### Follow-up study visits

**Figure 2.**
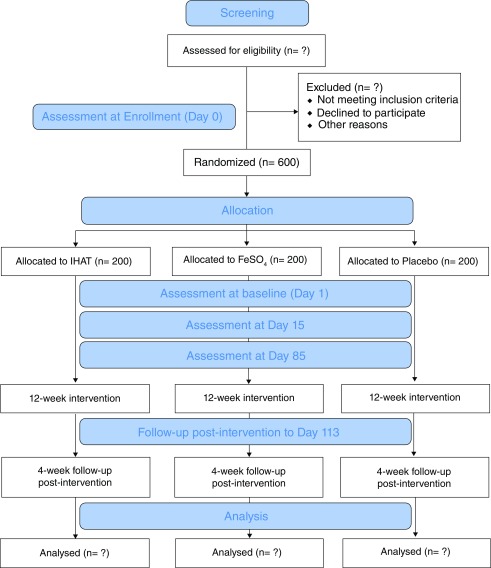
Consort 2010 flow diagram.

A flow chart of the study participant timeline is presented in Figure 2. On study Day 1, i.e. the first day of supplementation, each child is invited back to the study clinic and a photo is taken (with consent) for a study ID card that also contains the child’s randomisation/study ID number. At this visit, demographic and immunisation data are collected and the study morbidity questionnaire is completed ( Table 1). A venous blood sample and a stool sample are also collected (study baseline samples). After blood collection, the field staff give the child their allocated study arm supplement.

**Table 1.** Overview of the study morbidity questionnaire.

Category	Questions
Diarrhoea	Any diarrhoea? If yes, duration (days) If yes, number of stools per day If yes, is there blood/mucus in stool? Is the child urinating less? Is the child lethargic or unconscious? Is the child restless and irritable? Does the child have sunken eyes? Is the child drinking poorly? Is the child thirsty, drinking eagerly? Does the skin pinch go back slowly?
Fever	Axillary temperature History of fever? If yes, number of days
Cough	Cough? If yes, duration of cough (days)
Vomiting	Has the child been vomiting since last visit? If yes, number of days
Appetite	How is your child’s appetite? If decreased, number of days If decreased, describe
Medication	Has the child been or is on any medication since last visit? If yes, please state the medication
Other	Difficulty breathing? Convulsion? Has the child had any other illness since last visit? If yes, describe

Every day over the following 12 weeks, the field staff visits the child at home in order to administer the allocated study supplement and to check on the child’s health status. They complete the study morbidity questionnaire three times per week, which includes questions regarding fever, diarrhoea, vomiting, malaria symptoms, other illness, hospitalisation, appetite and medication. If a child is found unwell they are referred to the study nurse or clinician for evaluation and treatment. These check-ups continue 4 weeks post-supplementation to follow-up on adverse events.

Each week during the study supplementation period, children are invited back to the study clinic for a check-up by the study nurse and a finger prick to determine their malaria and Hb status. Children found with a positive RDT at any time during the supplementation period are further tested with a blood film and treated according to The Gambian national guidelines if malaria is confirmed. Any child where Hb falls below 7 g/dl during the supplementation period, discontinues the study supplementation and is offered standard-of-care oral iron treatment according to the national guidelines. These children continue to be followed up by the clinical team at the weekly clinics and will not be excluded from the data analysis but considered as treatment failures.

On study days 15 and 85, the child visits the study clinic and stool and venous blood samples are collected. Immunisation data is also recorded on these occasions. Height and weight is re-measured on study Day 85.

Venous blood samples are collected in S-Monovette® blood collection tubes either before the first meal of the day or, when this is not possible, at least 1 hour after the last meal. All blood samples are kept cool before transport to the Basse laboratory for processing. Stool samples are collected at home in toilet pots provided by the study team and lined with a disposable plastic liner at study days 1, 15 and 85. Stool samples can be collected within 7 days of the study time-point when it becomes difficult to collect the sample on that exact day, i.e. if the child travels or is not able to pass stool. The stool samples are aliquoted by the field staff as soon as possible after collection into a sterile Sarstedt stool collection tube and an OMNIgene® GUT sample collection kit (DNA Genotek).

At the end of the study follow-up period (i.e. Day 113), the children in any arm who still have anaemia (Hb<11 g/dl) are provided with standard-of-care iron supplementation for three consecutive months as per Gambian national and WHO guidelines.

Standardised protocols have been developed and are being used by all trial staff for conducting all field, laboratory and data procedures employed in this study. All deviations to these procedures is promptly reported following the study sponsor procedure and ICH-GCP guidelines. These methods can be made available upon request.

### Outcomes

All participants are assessed at three different time-points: at baseline (Day 1 of supplementation), and at 15 and 85 days after start of supplementation. The Day 15 time point will provide an indication of acute compound-related effects and the Day 85 time-point will be indicative of chronic (i.e. longer term) compound-related effects, for all the ‘safety’ outcomes.

The primary efficacy outcome of the trial is the proportion of children in each arm who resolve iron deficiency and either achieve a normal Hb or show an increase of at least 1 g/dl after 12 weeks of supplementation (i.e. at study Day 85). The primary safety outcome is the burden of moderate-severe diarrhoea. Therefore, there are four primary endpoints of the trial: (1) iron deficiency at Day 85; (2) Hb levels at Day 85; (3) ‘incidence density’ of moderate-severe diarrhoea over the 12 weeks (i.e. the number of new moderate-severe diarrhoea episodes per child over the 12 weeks supplementation period); (4) ‘period prevalence’ of moderate-severe diarrhoea over the 12 weeks supplementation period (i.e. the proportion of children with at least one episode of moderate-severe diarrhoea in this period). To assess iron deficiency, we will use regression-adjusted ferritin concentration below 12 μg/l, where ferritin values are adjusted for inflammation using the regression model recommended by the Biomarkers Reflecting Inflammation and Nutritional Determinants of Anemia (BRINDA) group ^6^.

Iron deficiency and Hb levels at Day 85 will be used to assess non-inferiority of IHAT relative to FeSO _4_ in terms of efficacy at treating IDA. We will determine the proportion of children in each arm who resolve iron deficiency and either achieve a normal Hb (≥ 11 g/dl) or an increase in Hb of at least 1 g/dl after 12 weeks of iron supplementation.

‘Incidence density’ and ‘period prevalence’ of moderate-severe diarrhoea will both be used to assess superiority of IHAT relative to FeSO _4_ and non-inferiority relative to placebo for the diarrhoea outcome (i.e. safety or tolerability). Moderate-severe diarrhoea is defined as those diarrhoea episodes where: (i) the child passes more than five loose or liquid stools per day, (ii) there is blood or mucus in the stool (dysentery), or (iii) the child shows signs of clinical dehydration (assessed by the study nurse based on physical signs such as little or no urination, sunken eyes, and skin that lacks its normal elasticity).

Secondary endpoints are faecal microbiome diversity and profile (particularly in terms of abundance of *Enterobacteriaceae*, *Bifidobacteriaceae, Lactobacillaceae*), abundance of enteric pathogens, faecal calprotectin (marker of gut inflammation), hospitalisation and morbidity (data collected three times per week using the study questionnaire), malaria infection (data collected every week with RDT), treatment failures (i.e. the number of children who have to discontinue study supplementation because their Hb falls below 7 g/dl), the proportion of days a child has diarrhoea over the intervention period (‘longitudinal prevalence’ of diarrhoea), the proportion of days a child has moderate-severe diarrhoea over the intervention period (‘longitudinal prevalence’ of moderate-severe diarrhoea), ‘incidence density’ of bloody diarrhoea (i.e. the number of bloody diarrhoea episodes per child-month of observation), markers of systemic inflammation (serum C-reactive protein (CRP) and alpha 1-acid glycoprotein (AGP)), and systemic markers of iron status (hepcidin, soluble transferrin receptor (sTfR), transferrin saturation and circulating non-transferrin bound iron - NTBI).

### Laboratory evaluations

Unless otherwise stated, all laboratory evaluations are carried out at the laboratories of the MRCG.

***Blood samples***. In the venous blood samples collected on study Days 1, 15 and 85, the following parameters are assessed: full haematology panel (using a Medonic ^TM^ Haematology Analyzer M-Series, Boule Diagnostics AB); serum ferritin, sTfR, serum iron and total iron binding capacity (for calculating transferrin saturation), CRP, AGP (using a fully automated biochemistry analyser Cobas Integra 400 plus); serum hepcidin (using the DRG® Hepcidin 25 (bioactive) HS ELISA); serum NTBI (using a fluorescent beads assay ^39^, at an external laboratory in the U.K.). In the weekly finger prick blood samples, Hb is determined using a HemoCue® Hb 301 system and RDT is performed using the SD Bioline Malaria Ag *P.f.* test (Standard Diagnostics, Inc.), these evaluations are conducted at the field study clinics.

***Stool samples***. Stool samples collected in the OMNIgene® GUT tube contain a DNA stabilising agent that ensures that samples can be kept at ambient temperature for 60 days. Total stool DNA is extracted from these samples using the Mo Bio PowerLyzer® PowerSoil® DNA Isolation Kit (Qiagen) within 6 weeks of sample collection. Stool DNA will be used for microbiome 16S rRNA sequencing (Illumina MiSeq platform), which will be conducted at an external laboratory in the U.K., and also for targeted qPCR of enteric pathogens.

In the stool samples collected in the Sarstedt stool collection tube the following parameters are assessed: calprotectin (using the Bühlmann fCAL® ELISA) and helminth egg count (using the Kato-Katz method ^40^).

### Data collection and data management

A password-protected study database has been established using the Research Electronic Data Capture (REDCap) secure web-based application based in PHP and MySQL. Access to the database is only given to members of the study team and the trial monitors and the database is managed by a dedicated data manager. All field data is captured in electronic case report forms (eCRF) and entered directly into handheld tablets (Samsung Galaxy Tab A (2016) SM-T580) using the Android 7.0 software and the REDCap mobile app. Data is synced daily into the database via secure Wi-Fi network.

The following data is recorded: date of informed consent, personal data (ID, initials, date of birth), socioeconomic data, height and weight, information on health status and regarding participation in other studies, date and time of all venous and finger prick blood collections, date and time of all faecal sample collections, date and time of supplement administration, data on morbidity, adverse events, lab results.

### Sample size

The hypotheses being tested in this study are:

1. Non-inferiority of IHAT compared to ferrous sulphate for correction of iron deficiency and either achieving a normal Hb or an increase of at least 1 g/dl after 12 weeks of iron supplementation. A non-inferiority margin of 0.1 is used. The null hypothesis is therefore the probability in the IHAT arm minus the response probability in the ferrous sulphate arm is less than or equal to –0.1.
2. Superiority of IHAT compared to ferrous sulphate in terms of incidence density of moderate-severe diarrhoea. Here the null hypothesis is that the mean number of new episodes in the IHAT arm is greater than or equal to the mean number in the ferrous sulphate arm.
3. Superiority of IHAT compared to ferrous sulphate in terms of prevalence of moderate-severe diarrhoea. Here the null hypothesis is that the prevalence of diarrhoea in the IHAT arm is greater than or equal to the prevalence in the ferrous sulphate arm.
4. Non-inferiority of IHAT compared to placebo in terms of prevalence of moderate-severe diarrhoea. Here the null hypothesis is that the prevalence of diarrhoea in the placebo arm minus the prevalence in the IHAT arm is less than or equal to – 0.1.

Because this is a preliminary study, with any significant results being retested in a subsequent pivotal study, we do not adjust for multiple testing. Each hypothesis is to be tested at a 10% one-sided type I error rate.

A sample size of 600 subjects completed equally randomised between the three arms, provides high power for each of the four hypotheses:

1. An 89% power to test non-inferiority on the IDA response probability, with a non-inferiority margin of 0.1 (0.583 on the odds ratio scale) assuming that the true response probability is 0.3 on IHAT and ferrous sulphate ^10, 13, 41^ arms.
2. A 90% power to test superiority for the incidence density of moderate-severe diarrhoea outcome assuming IHAT provides a 20% reduction in the mean for ferrous sulphate (i.e. from 1.28 episodes per child over the 12 weeks supplementation period to 1.02; calculations based on unpublished data for studies with iron supplements in rural Gambia).
3. A 90% power to test reduction in prevalence of moderate-severe diarrhoea from 25% in the ferrous sulphate arm to 15% in the IHAT arm.
4. A 93% power to test non-inferiority (0.1 non-inferiority margin, 0.583 on the odds ratio scale) of IHAT against placebo for prevalence of moderate-severe diarrhoea when both have 15% prevalence. For these calculations, we used published diarrhoea period prevalence data from studies with iron supplementation ^13, 17^.

For the secondary outcomes, the trial (n=200 per arm) will have over 85% power to detect significant differences between all the arms in terms of enterobacteria ^17^, NTBI ^39^ and calprotectin ^17^.

To account for a non-completion rate of ca. 15%, the total number of children we plan to enrol in the study is n=705.

### Statistical analysis

A full statistical analysis plan will be finalised prior to database lock and before breaking the randomisation code. We summarise briefly the planned statistical analysis here.

For the IDA response and diarrhoea prevalence outcomes, we will fit a logistic regression, adjusting for the strata created by age and Hb level groups prior to enrolment. For non-inferiority comparisons, the odds ratio for effect of IHAT relative to the relevant comparator arm will be estimated, and if the lower 90% one-sided confidence interval is above 0.583 (equivalent to the 10% non-inferiority margin above), non-inferiority will be declared for IHAT. For superiority comparisons, the data will be analysed in a similar way but superiority will be declared if the one-sided p-value for the Wald test of the effect of IHAT is less than 0.1.

For the diarrhoea incidence endpoint, a Poisson regression with treatment arm as predictor will be fitted to the data adjusting for the strata.

For the primary non-inferiority hypothesis, we will conduct per-protocol and intention-to-treat analyses and for the superiority hypotheses we will conduct intention-to-treat analyses.

The secondary endpoints will be tested using linear regression with the treatment arm, age and Hb level as covariates.

Multiple imputation will be used to account for missing data if there are missing covariates in more than 5% of participants. If there is substantial difference in the loss-to-follow-up rate in the different arms we will consider applying simple sensitivity analyses to investigate the robustness of results.

### Ethics statement

This study is being conducted in accordance with the ethical principles that have their origin in the Declaration of Helsinki, and that are consistent with the ICH GCP requirements, and in keeping with local regulatory requirements.

Scientific advice on the study protocol has been given by the UK Medicines and Healthcare products Regulatory Agency (MHRA 1400, 21/12/2016). The study protocol and any subsequent amendments have been reviewed and approved by The Gambia Government/MRC Joint Ethics Committee (reference SCC1489). Clinical Trials Authorisation has been granted by the Medicines Control Agency, The Gambia (HP373/347/16/MJK(80)).

This paper is written following the SPIRIT 2013 guidelines ^42^ ( Supplementary File 2). The trial is registered in ClinicalTrials.gov, identifier: NCT02941081 (registration date: 21 Oct 2016; https://clinicaltrials.gov/ct2/show/NCT02941081).

### Data and safety monitoring

An independent local safety monitor (LSM) and an independent Data and Safety Monitoring Board (DSMB) have been appointed to monitor quality control of the data, progress of recruitment and safety aspects of the IHAT-GUT trial, including the regular review of all adverse events. The LSM reviews adverse events monthly and the DSMB reviews adverse events quarterly during the study. Any serious adverse events are reported in real time to both the LSM and the DSMB.

No interim analysis will be conducted unless requested by the DSMB in response to adverse events. By August 2018, no interim analysis had been requested by the DSMB and the most recent recommendation was for the study to continue as planned.

### Trial monitoring

Trial oversight is provided by the Sponsor through the Clinical Trials Department at MRCG. An independent trial monitor regularly reviews good clinical practice (GCP) compliance, quality of data collection, sample analysis and the progress of the trial.

### Informed consent

All field workers taking part in the recruitment of participants have received GCP and informed consent training prior to study start. These field workers have also been trained on translating the contents of the information sheet into the different local languages. The field staff explain details of the study to illiterate parents in a language they understand, in the presence of an impartial literate witness, and in a room which ensures adequate privacy. The literate parents are allowed to read the information sheet in their own time. Parents are given enough time to ask questions and decide if they want their child to participate. No child starts any study specific procedure before informed consent is obtained. Participants will not get any remuneration for taking part in the study but will have access to free medical care for the duration of the study. Participants are protected in accordance with the study Sponsor Clinical Trial/Non Negligent Harm Insurance and Medical Malpractice Insurance.

### Confidentiality

Each participant is allocated an individual identification (ID) number, which is used to label all samples collected for the study and on the eCRF during the course of the study. All data is linked-anonymised and the linkage to the ID is not be possible without a lookup table, which is held only by the data manager and designated data staff during the course of the study. Once data collection is complete, analysis will be performed on an anonymised copy of the dataset. At all stages, staff/collaborators responsible for sample analysis will be blinded as to the subject’s identification. Together, these processes will ensure complete confidentiality of the data gathered and impartiality of data analysis.

### Dissemination and data access

Results of the study will be submitted for publication in relevant peer-reviewed open-access journals and key findings presented at international scientific meetings. The main findings will be disseminated to the National Nutrition Agency and the Ministry of Health in The Gambia. If this study suggests a possible benefit of IHAT above ferrous sulphate, contact will be made with key international organizations (e.g. WHO, UNICEF) and funding bodies to plan future pivotal trials. Any request for use of study data will have to be approved by the study Sponsor and the Ethics Committee. Data will only be made available in an anonymous format to external users. Data sharing will be in agreement with the Sponsor policy on research data sharing and with the Bill & Melinda Gates Foundation Global Access requirements.

### Study status

Screening for enrollment started in November 2017 and by August 2018, 645 subjects have been successfully recruited and randomised. The first patient first visit (Day 1) was on January 8, 2018. Field data collection will be completed in December 2018. Analysis, and submission for publication, of primary endpoint results will be completed by March 2019. The current protocol version is SCC1489 V5.0, 21 May 2018.

## Discussion

### Rationale for enrolling young children as the study population group

Young children living in resource-poor rural areas in sub-Saharan Africa, where there is high burden from diarrhoeal diseases, are one of the main population groups in need of safer forms of iron supplementation. Randomised-controlled clinical trials conducted in nearly 10,000 young children living in developing countries have consistently shown that current forms of iron supplements are associated with increased infection, including bloody diarrhoea ^10, 13, 16, 17^, and detrimental changes to the gut microbiome and gut inflammation ^17, 18, 43^, further increasing the burden from enteric infection and environmental enteropathy (i.e. persistent gut damage and inflammation that leads to malabsorption), which is a major cause of growth failure in children in resource-poor environments ^21, 22^. Furthermore, it appears that these effects are much more relevant in those resource-poor countries where enteric infection risk is higher and in the pre-school age group, since in older South-African children, with a low enteropathogen burden, iron supplementation does not appear to significantly affect the dominant bacterial groups in the colon or to increase gut inflammation ^44^.

If IHAT shows a benefit in the proposed trial, where it will be tested in the population group that is most sensitive to the gastrointestinal adverse effects of iron supplementation, then we believe it can be effective in any other population group. However, the reverse is not true, if IHAT was tested and found to be effective in adults or older children, it would not mean it would work in the young children group, since these have the most immature guts where the microbiome is not stable and where the mucosa is more susceptible ^45– 48^.

The health of all children in our study is monitored daily, as are all adverse events, including all diarrhoea episodes, malaria and other co-infections. Thus, ethically we are not subjecting children to any unnecessary risk or, in fact, any added risk beyond that expected with current iron supplements when used as per national guidelines.

### Rational for inclusion of a placebo group

Due to the possible detrimental effect of iron supplements in pre-school age children, particularly those living in resource-poor settings ^10, 13, 16– 18, 43^, there is now a strong rationale for a ‘no iron’ (placebo) control group thus leading to ethical equipoise. Furthermore, in these settings, there have been numerous randomised-controlled trials with iron supplementation in young children that have failed to show a significant decrease in anaemia prevalence or a clinically relevant increase in haemoglobin after intervention with iron supplements ^10, 13, 41^, as such the active control or standard treatment does not have proven efficacy in this setting. Furthermore, in The Gambia there is no national screening program or mandatory iron supplementation or home fortification policy for children, even though the prevalence of anaemia in this age group is extremely high ^35^. 

Therefore, inclusion of a placebo arm in this trial adheres to the guidelines for non-inferiority trials as stipulated in the ICH guidance *Choice of Control Group and Related Issues in Clinical Trials (ICH E10)* stating that “ *where there is no serious harm, such as death or irreversible morbidity in the study population, it is generally considered ethical to ask patients to participate in a placebo-controlled trials, even if they may experience discomfort as a result, provided the setting is non-coercive and patients are fully informed about available therapies and the consequences of delaying treatment”* ^49^.

### Rational for treatment effect sizes

Because many trials conducted in children from resource-poor countries with ferrous sulphate or ferrous fumarate supplementation have still failed to provide anaemia resolution in interventions shorter than 6 months, we considered that an increase of 1 g/dl in haemoglobin after 3 months is an indication of treatment efficacy. We have based our assay sensitivity on data published with relevant iron supplement studies in children available in the literature ^10, 13, 41^. Nonetheless, we plan to also use the data collected in this study to conduct an exploratory analysis for assay sensitivity, using the comparisons between the active control and the placebo arms to determine the true effect size of iron supplementation in this population group. This is important since assay sensitivity is an essential property of a non-inferiority trial, allowing us to determine if both iron supplements are effective or if neither was effective as per FDA guidelines ^50^. These data will be very useful so that adequate treatment effect sizes can be used to power any future studies, in particular a pivotal trial, both for ferrous sulphate and IHAT supplementation effects.

Additionally, efficacy comparisons with the placebo group will tell us if iron supplements are preventing haemoglobin from decreasing further and preventing severe anaemia as children grow, which we consider to be a positive effect, even when overall anaemia prevalence may not decrease for this age group due to the high demands for iron during fast growth.

In terms of safety, the placebo arm will also allow us to rule-out any negative impact of IHAT on diarrhoea episodes, the gut microbiome and infection risk.

### Mitigation of risk of malaria and co-infections

There are risks associated with a large intake of iron supplements, especially in areas of malaria endemicity. The dose of iron given daily in the reference arm (12.5 mg) is the recommended by WHO for this age group in non-malarious areas or malaria-endemic areas where it should be implemented in conjunction with measures to prevent, diagnose and treat malaria and co-infections ^34^. The iron dose in the IHAT arm (20 mg) is the bioequivalent dose, i.e. the same absolute amount of iron should be absorbed as in the ferrous sulphate arm, and because IHAT should be safer to the gut, the unabsorbed fraction should not cause detrimental effects, such as infectious diarrhoea. This dose (20 mg Fe) is still less than the new WHO recommendation for children in the 24–35 month age group (i.e. 30 mg, ^34^).

In any case, in this study with the daily visits by the field staff and the weekly visits to the study clinics, all children are closely monitored and at the first sign of infection or malaria they are referred to the study nurse or clinician for clinical assessment and treatment as per clinical guidelines.

## Conclusions

This study will provide the first clinical trial data for IHAT, an innovative nano-iron supplement, and will enable us to obtain high-quality data for its safety and efficacy in correcting iron deficiency and improving anaemia in sub-Saharan African children, the population group most in need of a better oral iron supplement.

The trial will also contribute to our better understanding of iron deficiency and anaemia treatment effect sizes with the current gold-standard of iron supplementation, and to our understanding of how iron supplementation and iron deficiency impact on enteric infection and diarrhoea risk.

If IHAT is successful in this trial, these data will provide the evidence needed to encourage further development so that IHAT can be implemented as a novel iron source for use in micronutrient intervention strategies aimed at children and women living in resource-poor countries and, hence, help to reduce the global burden of IDA. We envisage that a future phase III pragmatic trial would test efficacy and safety of IHAT, used as part of multi-micronutrient supplements, in children and pregnant women across multiple countries.

## Data availability

No data is associated with this article.

## Supplementary material

Supplementary File 1: Participant information sheet and consent form.

.

Supplementary File 2: Completed SPIRIT checklist.

## References

[1] GBD 2016 Disease and Injury Incidence and Prevalence Collaborators: Global, regional, and national incidence, prevalence, and years lived with disability for 328 diseases and injuries for 195 countries, 1990-2016: a systematic analysis for the Global Burden of Disease Study 2016. *Lancet.* 2017;390(10100):1211–1259. 10.1016/S0140-6736(17)32154-2 28919117 PMC5605509

[2] Global Burden of Disease Study: Global Burden of Disease Study 2016 (GBD 2016) Results. In. Seattle, United States: Institute for Health Metrics and Evaluation (IHME);2016.

[3] Stevens GA Finucane MM De-Regil LM: Global, regional, and national trends in haemoglobin concentration and prevalence of total and severe anaemia in children and pregnant and non-pregnant women for 1995–2011: a systematic analysis of population-representative data. *Lancet Glob Health.* 2013;1(1):e16–e25. 10.1016/S2214-109X(13)70001-9 25103581 PMC4547326

[4] WHO: The global prevalence of anaemia in 2011. In. Geneva: World Health Organization;2015 Reference Source

[5] Wirth JP Woodruff BA Engle-Stone R: Predictors of anemia in women of reproductive age: Biomarkers Reflecting Inflammation and Nutritional Determinants of Anemia (BRINDA) project. *Am J Clin Nutr.* 2017;106(Suppl 1):416S–427S. 28615262 10.3945/ajcn.116.143073 PMC5490645

[6] Engle-Stone R Aaron GJ Huang J: Predictors of anemia in preschool children: Biomarkers Reflecting Inflammation and Nutritional Determinants of Anemia (BRINDA) project. *Am J Clin Nutr.* 2017;106(Suppl 1):402S–415S. 28615260 10.3945/ajcn.116.142323 PMC5490650

[7] Kassebaum NJ Jasrasaria R Naghavi M: A systematic analysis of global anemia burden from 1990 to 2010. *Blood.* 2014;123(5):615–624. 10.1182/blood-2013-06-508325 24297872 PMC3907750

[8] Stoltzfus RJ Mullany L Black RE: Iron deficiency anaemia. In: *Comparative quantification of health risks: global and regional burden of disease attributable to selected major risk factors.*edn. Edited by Ezzati M, Lopez AD, Rodgers A, Murray CJL. Geneva: WHO;2004;163–209. Reference Source

[9] Sazawal S Black RE Ramsan M: Effects of routine prophylactic supplementation with iron and folic acid on admission to hospital and mortality in preschool children in a high malaria transmission setting: community-based, randomised, placebo-controlled trial. *Lancet.* 2006;367(9505):133–143. 10.1016/S0140-6736(06)67962-2 16413877

[10] Soofi S Cousens S Iqbal SP: Effect of provision of daily zinc and iron with several micronutrients on growth and morbidity among young children in Pakistan: a cluster-randomised trial. *Lancet.* 2013;382(9886):29–40. 10.1016/S0140-6736(13)60437-7 23602230

[11] Prentice AM Verhoef H Cerami C: Iron fortification and malaria risk in children. *JAMA.* 2013;310(9):914–915. 10.1001/JAMA.2013.6771 24002276 PMC6136145

[12] Prentice AM: Iron metabolism, malaria, and other infections: what is all the fuss about? *J Nutr.* 2008;138(12):2537–2541. 10.3945/jn.108.098806 19022986

[13] Zlotkin S Newton S Aimone AM: Effect of iron fortification on malaria incidence in infants and young children in Ghana: a randomized trial. *JAMA.* 2013;310(9):938–947. 10.1001/jama.2013.277129 24002280

[14] Prentice AM Mendoza YA Pereira D: Dietary strategies for improving iron status: balancing safety and efficacy. *Nutr Rev.* 2017;75(1):49–60. 10.1093/nutrit/nuw055 27974599 PMC5155616

[15] Tolkien Z Stecher L Mander AP: Ferrous sulfate supplementation causes significant gastrointestinal side-effects in adults: a systematic review and meta-analysis. *PLoS One.* 2015;10(2):e0117383. 10.1371/journal.pone.0117383 25700159 PMC4336293

[16] Mayo-Wilson E Imdad A Junior J: Preventive zinc supplementation for children, and the effect of additional iron: a systematic review and meta-analysis. *BMJ Open.* 2014;4(6):e004647. 10.1136/bmjopen-2013-004647 24948745 PMC4067863

[17] Jaeggi T Kortman GA Moretti D: Iron fortification adversely affects the gut microbiome, increases pathogen abundance and induces intestinal inflammation in Kenyan infants. *Gut.* 2015;64(5):731–42. 10.1136/gutjnl-2014-307720 25143342

[18] Zimmermann MB Chassard C Rohner F: The effects of iron fortification on the gut microbiota in African children: a randomized controlled trial in Cote d'Ivoire. *Am J Clin Nutr.* 2010;92(6):1406–1415. 10.3945/ajcn.110.004564 20962160

[19] Tang M Frank DN Sherlock L: Effect of Vitamin E With Therapeutic Iron Supplementation on Iron Repletion and Gut Microbiome in US Iron Deficient Infants and Toddlers. *J Pediatr Gastroenterol Nutr.* 2016;63(3):379–385. 10.1097/MPG.0000000000001154 27548249 PMC4994979

[20] Paganini D Uyoga MA Zimmermann MB: Iron Fortification of Foods for Infants and Children in Low-Income Countries: Effects on the Gut Microbiome, Gut Inflammation, and Diarrhea. *Nutrients.* 2016;8(8): pii: E494. 10.3390/nu8080494 27529276 PMC4997407

[21] Naylor C Lu M Haque R: Environmental Enteropathy, Oral Vaccine Failure and Growth Faltering in Infants in Bangladesh. *EBioMedicine.* 2015;2(11):1759–1766. 10.1016/j.ebiom.2015.09.036 26870801 PMC4740306

[22] Lin A Arnold BF Afreen S: Household environmental conditions are associated with enteropathy and impaired growth in rural Bangladesh. *Am J Trop Med Hyg.* 2013;89(1):130–137. 10.4269/ajtmh.12-0629 23629931 PMC3748469

[23] Pasricha SR Armitage AE Prentice AM: Reducing anaemia in low income countries: control of infection is essential. *BMJ.* 2018;362:k3165. 10.1136/bmj.k3165 30068664

[24] Powell JJ Bruggraber SF Faria N: A nano-disperse ferritin-core mimetic that efficiently corrects anemia without luminal iron redox activity. *Nanomedicine.* 2014;10(7):1529–1538. 10.1016/j.nano.2013.12.011 24394211 PMC4315135

[25] Pereira DI Mergler BI Faria N: Caco-2 cell acquisition of dietary iron(III) invokes a nanoparticulate endocytic pathway. *PLoS One.* 2013;8(11):e81250. 10.1371/journal.pone.0081250 24278403 PMC3836913

[26] Theil EC Chen H Miranda C: Absorption of iron from ferritin is independent of heme iron and ferrous salts in women and rat intestinal segments. *J Nutr.* 2012;142(3):478–483. 10.3945/jn.111.145854 22259191 PMC3278266

[27] Kalgaonkar S Lonnerdal B: Receptor-mediated uptake of ferritin-bound iron by human intestinal Caco-2 cells. *J Nutr Biochem.* 2009;20(4):304–311. 10.1016/j.jnutbio.2008.04.003 18602806 PMC2684700

[28] San Martin CD Garri C Pizarro F: Caco-2 intestinal epithelial cells absorb soybean ferritin by mu2 (AP2)-dependent endocytosis. *J Nutr.* 2008;138(4):659–666. 10.1093/jn/138.4.659 18356317 PMC3065195

[29] Pereira DI Aslam MF Frazer DM: Dietary iron depletion at weaning imprints low microbiome diversity and this is not recovered with oral Nano Fe(III). *MicrobiologyOpen.* 2015;4(1):12–27. 10.1002/mbo3.213 25461615 PMC4335973

[30] Pereira DI Bruggraber SF Faria N: Nanoparticulate iron(III) oxo-hydroxide delivers safe iron that is well absorbed and utilised in humans. *Nanomedicine.* 2014;10(8):1877–1886. 10.1016/j.nano.2014.06.012 24983890 PMC4228177

[31] Aslam MF Frazer DM Faria N: Ferroportin mediates the intestinal absorption of iron from a nanoparticulate ferritin core mimetic in mice. *FASEB J.* 2014;28(8):3671–3678. 10.1096/fj.14-251520 24776745 PMC4101650

[32] Pan YH Sader K Powell JJ: 3D morphology of the human hepatic ferritin mineral core: new evidence for a subunit structure revealed by single particle analysis of HAADF-STEM images. *J Struct Biol.* 2009;166(1):22–31. 10.1016/j.jsb.2008.12.001 19116170 PMC2832756

[33] Latunde-Dada GO Pereira DI Tempest B: A nanoparticulate ferritin-core mimetic is well taken up by HuTu 80 duodenal cells and its absorption in mice is regulated by body iron. *J Nutr.* 2014;144(12):1896–1902. 10.3945/jn.114.201715 25342699 PMC4230207

[34] WHO: Guideline: Daily Iron Supplementation in Infants and Children. In. Geneva, Switzerland: World Health Organization;2016. 27195348

[35] The Gambia Bureau of Statistics (GBOS), ICF International: The Gambia Demographic and Health Survey 2013. In. Banjul, The Gambia, and Rockville, Maryland, USA: GBOS and ICF International;2014 Reference Source

[36] Saha D Akinsola A Sharples K: Health Care Utilization and Attitudes Survey: understanding diarrheal disease in rural Gambia. *Am J Trop Med Hyg.* 2013;89(1 Suppl):13–20. 10.4269/ajtmh.12-0751 23629926 PMC3748496

[37] Kotloff KL Nataro JP Blackwelder WC: Burden and aetiology of diarrhoeal disease in infants and young children in developing countries (the Global Enteric Multicenter Study, GEMS): a prospective, case-control study. *Lancet.* 2013;382(9888):209–222. 10.1016/S0140-6736(13)60844-2 23680352

[38] Pasricha SR Atkinson SH Armitage AE: Expression of the iron hormone hepcidin distinguishes different types of anemia in African children. *Sci Transl Med.* 2014;6(235):235re3. 10.1126/scitranslmed.3008249 24807559

[39] Ma Y Podinovskaia M Evans PJ: A novel method for non-transferrin-bound iron quantification by chelatable fluorescent beads based on flow cytometry. *Biochem J.* 2014;463(3):351–362. 10.1042/BJ20140795 25093426

[40] WHO Expert Committee: Prevention and control of schistosomiasis and soil-transmitted helminthiasis. *World Health Organ Tech Rep Ser.* 2002;912:i–vi, 1–57. 12592987

[41] De-Regil LM Suchdev PS Vist GE: Home fortification of foods with multiple micronutrient powders for health and nutrition in children under two years of age. *Cochrane Database Syst Rev.* 2011; (9):CD008959. 10.1002/14651858.CD008959.pub2 21901727

[42] Chan AW Tetzlaff JM Gøtzsche PC: SPIRIT 2013 explanation and elaboration: guidance for protocols of clinical trials. *BMJ.* 2013;346:e7586. 10.1136/bmj.e7586 23303884 PMC3541470

[43] Paganini D Uyoga MA Kortman GAM: Prebiotic galacto-oligosaccharides mitigate the adverse effects of iron fortification on the gut microbiome: a randomised controlled study in Kenyan infants. *Gut.* 2017;66(11):1956–1967. 10.1136/gutjnl-2017-314418 28774885

[44] Dostal A Baumgartner J Riesen N: Effects of iron supplementation on dominant bacterial groups in the gut, faecal SCFA and gut inflammation: a randomised, placebo-controlled intervention trial in South African children. *Br J Nutr.* 2014;112(4):547–556. 10.1017/S0007114514001160 24916165

[45] Timmerman HM Rutten NBMM Boekhorst J: Intestinal colonisation patterns in breastfed and formula-fed infants during the first 12 weeks of life reveal sequential microbiota signatures. *Sci Rep.* 2017;7(1):8327. 10.1038/s41598-017-08268-4 28827640 PMC5567133

[46] Yatsunenko T Rey FE Manary MJ: Human gut microbiome viewed across age and geography. *Nature.* 2012;486(7402):222–227. 10.1038/nature11053 22699611 PMC3376388

[47] Lim ES Zhou Y Zhao G: Early life dynamics of the human gut virome and bacterial microbiome in infants. *Nat Med.* 2015;21(10):1228–1234. 10.1038/nm.3950 26366711 PMC4710368

[48] Houghteling PD Walker WA: Why is initial bacterial colonization of the intestine important to infants' and children's health? *J Pediatr Gastroenterol Nutr.* 2015;60(3):294–307. 10.1097/MPG.0000000000000597 25313849 PMC4340742

[49] Choice of control group and related issues in clinical trials E10. Reference Source 12356096

[50] Non-Inferiority Clinical Trials to Establish Effectiveness. Guidance for Industry. Reference Source

