# Supplementary material for: A novel nano-iron supplement to safely combat iron deficiency and anaemia in young children: The IHAT-GUT double-blind, randomised, placebo-controlled trial protocol
Source: Gates Open Res. 2018 Oct 11;2:48. Originally published 2018 Sep 21. [Version 2] doi: 10.12688/gatesopenres.12866.2 (PMC6266659; doi:10.12688/gatesopenres.12866.2)
Supplement: Supplementary file 1 [file gatesopenres-2-13967-s0000.tgz › 50aa276a-ac7b-4c95-99d0-1c672c7401a4_ICD_IHAT-Gut_V3_16_02_18.pdf]

PARTICIPANT INFORMATION SHEET

Version 3.0 Date 16th February 2018

Study Title: A novel nano-iron supplement (IHAT) to safely combat iron deficiency and anaemia (IDA) in young children: a double-blind randomised controlled trial.

IHAT-Gut

SCC:	1489	Protocol:	V 4.0

Sponsor & Funder: MRC Unit The Gambia at the London School of Hygiene & Tropical Medicine (sponsor) & Bill and Melinda Gates Foundation (funder)

What is informed consent?

You are invited to let your child take part in a research study. Before you decide, you need to understand why the research study is being done and what it will involve. Please take time to read the following information or get the information explained to you in your language. Listen carefully and feel free to ask if there is anything that is not clear or you do not understand. You may also wish to consult your spouse, family members, friends or others before deciding to let your child take part in the study.

If you decide to let your child join the study, you will need to sign or put a thumbprint on a consent form saying you agree for your child to be in the study. You will receive a copy of this.

Why is this study being done?

Anaemia (not enough iron in your blood) in young children is very common and affects more than 70% of all children under 5 y. in The Gambia. Iron supplementation (giving iron) is the recommended treatment to improve the child's defences against disease and their brain development. However, iron can cause intestinal symptoms such as diarrhoea and infection in very young children.

With this study we want to test a new compound that is similar to the iron found naturally in foods and that we believe will reduce the number of children with anaemia without increasing their risk of intestinal infection or diarrhoea. If our new compound is successful in this study, it could be used instead of other iron compounds to make giving iron safer to the gut.

The results of the study will be made available to your community.

What is the new drug?

The new iron supplement we are testing is called IHAT, which stands for iron hydroxide adipate tartrate. This compound has been in development at the MRC Unit in Cambridge for the last 11 years and it looks like the iron present in plant-based foods. We will compare the new compound

with ferrous sulphate, which is one of the most common iron supplements used in children, and with placebo (a sugar compound).

Each compound will be ingested every day with the amount of iron equivalent to the one recommended by the World Health Organization for infants and young children (12.5 mg of iron per day).

What does this study involve?

The following will be required from your child if you agree to take part:

Your child's health will be examined and height and weight will be measured. Then we will take 2 drops of blood from your child's finger to test if they are anaemic. If yes, we will draw a small amount of blood from your child (1 ml, which is less than half a teaspoon) to test if your child can participate in the study as only children with little iron in their body can participate in the study, as they will need extra iron. If yes, your child will then wait for around 4 weeks to take part in the study until we get all the blood results back from the lab. We will then take another 2 drops of blood from your child's finger to confirm they can still take part and your child will be randomly allocated to one of the three study groups:

Group A: Ferrous Sulphate 12.5 mg iron daily

Group B: IHAT equivalent to 12.5 mg iron daily

Group C: Placebo in the form of a sugar compound

A week later, will take a picture of your child and give you a study ID card for you to keep safe and also place a study wristband on your child's wrist that we ask you not to remove during the study. This wristband will not cause any discomfort to your child and it will help us identify to which of the three study groups your child is allocated. We will then draw some more blood from your child (5 ml, which is approximately one full teaspoon) and we will also ask you to collect a stool sample from your child. Your child will then take one of the compounds every day for 12 weeks and this will be given by a field assistant. The compounds will be given dissolved in a couple of spoonful of a sugar drink. The field assistant will additionally ask some questions regarding the health of your child three times per week.

On study day 15 and 85 we will again draw some blood from your child (5 ml, which is approximately one full teaspoon) and ask you to collect another stool sample from your child. We will monitor the health of your child and every week they will visit one of the study health facilities for a check-up where we will take another 2 drops of blood from your child's finger to test if they have malaria and see if they are still anaemic. On study day 85 (end of the study iron supplementation period) we will measure height and weight again.

In case the investigator discovers your child is sick and decides that he/she cannot participate in the study because of that, he/she will receive immediate care at the study site and then be referred to the appropriate health care centre.

If the study needs to be stopped, you will be informed and your child will have the normal medical care.

The field assistants will continue to visit your child daily for an extra 4 weeks after the end of the study iron supplementation period to check their health. At the end of the study all children who remain anaemic will be supplemented with iron according to the national guidelines.

What will happen to the samples taken in this study?

The collected blood and stool samples will be transported to our research laboratories in Basse and Keneba. There we will test if your child's blood is low in iron and if your child has an infection. We will also store the samples for further analysis of markers that may indicate side effects of iron supplements. Some of the blood and stool samples will be transferred to laboratories overseas for analysis because we don't have the equipment required for measuring all of the factors we are investigating in the Gambia.

What harm or discomfort can you expect in the study?

Collection of blood can cause minor discomfort, but will not cause any harm to your child. There is a small risk that iron can cause diarrhoea or other tummy discomfort. This will be closely monitored by our daily visits.

What benefits can you expect in the study?

By participating in this study your child will receive medical care at a level greater than that usually given within the study area. We will closely monitor your child's health, and give you regular feedback.

Will you be compensated for your child's/ward's participation in the study?

You will not get paid for your child taking part in the study, but you will get either transport by MRC Unit The Gambia at LSHTM or get the costs for the transport to the health centre reimbursed.

Are there other products or treatment?

No.

What happens if you refuse to participate in the study or change your mind later?

You are free to let your child take part or not in the study and you can refuse his/her participating at any time without giving a reason. This will not affect the medical care that your child would normally receive.

In case you decide to withdraw your child's participation during the study we will not work on your child's samples without your permission, but any information already generated from the samples until that time will be used and samples already collected, for which you have given consent, will also be analysed and data used. The study doctor may also ask for tests for your child's safety.

Should any new information become available during the study that may affect your child's participation, you will be informed as soon as possible.

What compensation will be available if your child is injured during the study?

We will be responsible to provide treatment to any injury caused by the research study through the London School of Hygiene and Tropical Medicine (LSHTM) clinical trial/non-negligent harm insurance and medical malpractice insurance. If your child has an unwanted reaction, we will treat him/her or refer him/her as needed.

If medical treatment is required as an emergency, please refer to your health centre or clinic and contact the field worker who gave his/her telephone number to you or contact Dr Mohammad Ilias Hossain on [+2207956138] or Dr Ogochukwu Ofordile on [+2207088830].

How your child's information will be kept and who will be allowed to see it?

All information that is collected about your child in the course of the study will be kept strictly confidential. Your child's personal information will only be available to the study team members and might be seen by some rightful persons from the Ethics Committee, Government authorities and sponsor.

Who should you contact if you have questions?

If you have any queries or concerns you can contact Dr Mohammad Ilias Hossain on [+2207956138] or Dr Ogochukwu Ofordile on [+2207088830] and you can always call the personal numbers of the study staff given to you. If you have any concerns you can also contact staff at your health centre or clinic.

Please feel free to ask any question you might have about the research study.

Who has reviewed this study?

This study has been reviewed and approved by a panel of scientists at the MRC Unit The Gambia at LSHTM and the Gambia Government/MRC Joint Ethics Committee, which consists of scientists and lay persons to protect your rights and wellbeing.

SCC:	1489	V 4.0

CONSENT FORM

Participant's Name ____

Participant's Identification Number: |__|__|__|__|__|__|__|__|__|__|

______________________________________ **OR** ______________________________________

(Printed name of parent) (Printed name of guardian)

☐ I have read the written information **OR**

☐ I have had the information explained to me by study personnel in a language that I understand

and I

- confirm that my choice to let my child participate is entirely voluntarily,
- confirm that I have had the opportunity to ask questions about this study and I am satisfied with the answers and explanations that have been provided,
- understand that I grant access to data about my child to authorised persons described in the information sheet,
- have received sufficient time to consider to let my child take part in this study
- agree to allow my child take part in this study.

Tick as appropriate

I agree for my child's samples to be shipped outside the Gambia. Yes ☐ No☐

I agree to further research on my child's samples as described in the information sheet. Yes ☐ No ☐

Participant's parent/guardian signature/thumbprint* [ ]

____

Date (dd/mmm/yyyy) Time (24hr)

Printed name of impartial witness* ______________________________________

Signature of impartial witness* ______________________ ______________________________________

Date (dd/mmm/yyyy) Time (24hr)

Printed Name of Person obtaining consent ______________________________________

I attest that I have explained the study information accurately in ______________________ to, and was understood to the best of my knowledge by, the participant/parent/guardian and that he/she has freely given consent to participate *in the presence of the above named impartial witness (where applicable).

Signature of Person obtaining consent ______________________ ______________________________________

Date (dd/mmm/yyyy) Time (24hr)

**Only required if the participant is unable to read or write.*

A copy of this informed consent document has been provided for the participant.
