# Supplementary material for: A novel nano-iron supplement to safely combat iron deficiency and anaemia in young children: The IHAT-GUT double-blind, randomised, placebo-controlled trial protocol
Source: Gates Open Res. 2018 Oct 11;2:48. Originally published 2018 Sep 21. [Version 2] doi: 10.12688/gatesopenres.12866.2 (PMC6266659; doi:10.12688/gatesopenres.12866.2)
Supplement: Peer review history

# Peer review: Reviewer response for version 2

**DOI:** 10.21956/gatesopenres.13967.r26711

**Published:** 2018-10-15

**Authors:** Toby Richards

**Affiliations:** 1 Division of Surgery and Interventional Science, University College London, London, UK

No further comments.

I have read this submission. I believe that I have an appropriate level of expertise to confirm that it is of an acceptable scientific standard.

# Peer review: Reviewer response for version 1

**DOI:** 10.21956/gatesopenres.13950.r26668

**Published:** 2018-10-04

**Authors:** Robert C. Hider

**Affiliations:** 1 Division of Pharmaceutical Science, King’s College London, London, UK

This article is very well planned and well resourced. The project is highly relevant to treatment of anemia. To provide a FeSO _4_ substitute of equivalent activity is an important advance. This is not glamorous work but the possible replacement of FeSO _4_ by an alternate iron source is extremely important, as compliance with FeSO _4_ can be poor.

 A relatively new development in this type of work is to study any changes induced in the gut flora. The results from this part of the proposed study will certainly be interesting, but may be difficult to interpret.

 The clinical trial includes a placebo as well as a FeSO _4_ control. This is important, as many such trials include one or the other, but not both. The direct comparison of the three different conditions will facilitate the interpretation of the clinical data.

I have read this submission. I believe that I have an appropriate level of expertise to confirm that it is of an acceptable scientific standard.

# Peer review: Reviewer response for version 1

**DOI:** 10.21956/gatesopenres.13950.r26671

**Published:** 2018-10-01

**Authors:** Toby Richards

**Affiliations:** 1 Division of Surgery and Interventional Science, University College London, London, UK

Many thanks for asking me to review this trial protocol. This trial offers a potential breakthrough to one of the biggest diseases worldwide.

 Iron deficiency is the most common nutritional disease worldwide. A disease that impacts people form countries of all GDP. Iron deficiency is the commonest cause of anaemia and associated with 8 years of living with disability. Iron deficiency and anaemia are so common they are hidden in plain sight.  

 In low income countries, the situation is worsened due to poor diet (low in meat, which has good iron absorption) and concurrent infections (which reduce iron absorption). Iron deficiency leads to childhood growth retardation and reduced cognitive development. In adults’ iron deficiency impacts mental and physical performance. In pregnancy the situation worsens with a higher risk of intrauterine growth retardation and death to both mother and baby at childbirth.

 A key problem to iron deficiency is lack of available good therapies. First line is dietary modification and then iron tablets. Iron tablets are essentially iron salts. The iron is poorly absorbed (only 5%) the remainder sits in the gut causing local toxicity, side effects and potentially exacerbating infection. An alternative is an iron infusion. This is simply not available or possible in many populations let alone in those countries who cannot afford healthcare.

 There is little funding to trials with simple and cheap solutions and no new oral iron preparations in the last decade. The Gates Foundation are to be applauded to support this work.

**Main Comments**

 Overall, this manuscript would benefit from being shortened and simplified. The message that this is a novel oral iron with a unique absorption pathway that may impact anaemia management without GI side effects does not come through. The authors should not under estimate the novelty of the invention of a nano-iron and how this works

 For a full protocol it can be difficult as there is so much information. The main issue is complexity of the primary endpoint. It is very unusual to have 4 primary endpoints. This leads to a lot of confusion in the hypothesis, aim(s) objectives and methods. Ideally this should be one key primary endpoint or in this case one efficacy and one safety endpoint. It is not a Multiarm multlistage or adaptive trial so randomising to 3 groups with 4 primary endpoints is difficult. Perhaps, broach this early on and explain (I suspect) the funder's and sponsors requirement for the additional endpoints, then refer to efficacy and safety endpoints moving forwards.

**Abstract**

 Please simplify and shorten and explain what IHAT is and why it is relevant:

 Oral iron is often ineffective as poorly absorbed and associate with gut side effects. IHAT is a novel nano-iron preparation with high efficacy and >50% absorption therefore overcoming these issues enabling lower dosage than conventional therapy with fewer side effects.

 Is this a Phase 2a? efficacy trial to test non-inferiority of IHAT to treat anaemia compared to FeSo4 and safety of IHAT in this population.

 Remove: Assessments are conducted at baseline, Day 15, and Day 85 of the supplementation period.

 Secondary objectives are to assess the superiority of IHAT to ferrous sulphate and non-inferiority to placebo in terms of side effects of diarrhoea.

 Secondary endpoints could be referred to mechanistic endpoints. Further, mechanistic assessments, to test whether IHAT reduces the burden of enteric pathogens, morbidity, and intestinal inflammation, and that it does not cause detrimental changes to the gut microbiome, particularly in relation to *Lactobacillaceae*, *Bifidobacteriaceae *and *Enterobacteriaceae*.

**Manuscript**

 Many readers will not know how iron is absorbed in the body so the authors are encouraged to simplify and explain the basic physiology of iron absorption, the problem of oral iron compounds and therefore the novel properties of IHAT.

**Introduction**

 The authors provide an overview of ID and Anaemia. However, data on YLD and DALY are from adults not specific to children. They then talk about data in children. Please add a sentence on how ID and anaemia impact childhood development and illness to bring these two points closer together.

 Also perhaps explain the difference between ID and Anaemia or simply refer to the problem as IDA throughout

 Before rationale and aims, introduce a further sub heading ‘the problem’ to highlight how normal iron supplements do not work due to poor absorption and GI side effect. This is a key point to why IHAT is needed and many readers simply will not know that of 200mg FeSo4 less that 5% is actually absorbed.

 In rationale, please reduce the finer details of the compound and increase the background on earlier preclinical studies. What have you shown so far?

 Please list the aim (correct hb) and objectives (infection / side effects) as a separate subheading for clarity (as above comments).

**Protocol**

 Hypothesis is clear.

 Remove comparator from this paragraph as that is a method.

 Normally there is one primary endpoint then a series of secondary endpoints. It is confusing with 4 objectives. Please clarify through the manuscript the aim and objectives, primary and secondary endpoints.

 Remove details of any follow up study as not relevant to this protocol?

**Study Design & Setting**

 Please use sub headings to the design and sponsorship etc. then Setting to highlight the population (move details on transport of samples to methods not setting)

**Criteria**

 Why exclude previous / current iron supplements – as if still have IDA then this is failure of therapy.

 Is haemoglobinopathy excluded?

 Childhood blood testing can be difficult – was this a criteria for inclusion i.e. a child must be well and able to give a venous blood sample?

**Intervention**

 Conducting, clinical trials in this setting is very difficult. There is clearly a labour intensive methodology. Has this been validated (i.e. are these standardised methods in this setting) please provide a reference.

 The trial preparations have been manufactured to enable concealment and therefor blinding of the children. Are there blinded and unblinded assessors?

 How is concealment bias ensured if there are weekly hb testing - are these results kept blinded also do all children attend or only some – will there be reporting bias as only the sicker children return for repeat testing?

 Will children and healthcare workers be asked if they have an ideal of what intervention was given to ensure lack of bias? It may be clear that a child’s health is improving and this may in turn lead to higher compliance compared to a group where the intervention is causing side effects.

**Outcomes**

 Resolving iron deficiency and anaemia correction are two separate endpoints. What is the definition of resolving iron deficiency and also for correcting anaemia. A patient can have no anaemia but be iron deficient and vice versa.

 It seems the endpoint is Hb correction but hb rise > 1g/dl is not the same as an absolute correction of anaemia. Should this endpoint be change in Hb?

 Again, having 4 primary endpoints is unusual and difficult to comprehend in light of the aim and objectives. Also the primary endpoint normally reflects the previous data, principle question and denominator of the power calculation. I suspect the funder/regulatory authority had input to this but I urge a more simplified primary question.

 If 705 to be consented and enrolled please use this number throughout for enrolment to the trial.

**Discussion**

 As many of the patients have been enrolled, can the authors please highlight any interim analysis and also (I assume) the DSMC have approved continuation.

 Overall this is a superb study but I urge the authors to simplify to two questions – does IHAT work and is it safe?

I have read this submission. I believe that I have an appropriate level of expertise to confirm that it is of an acceptable scientific standard, however I have significant reservations, as outlined above.

# Response

**Published:** 2018-10-06

**Authors:** Dora Pereira

**Affiliations:** University of Cambridge, UK

Many thanks for your valuable comments and for reviewing this protocol. We hope we have address your reservations in the revised manuscript.

Here are some specific responses to some your comments.

 

- *The authors should not under estimate the novelty of the invention of a nano-iron and how this works*

 

We have tried to highlight better the novelty of IHAT and why it should work better than current oral iron.  

 

- *The main issue is complexity of the primary endpoint. It is very unusual to have 4 primary endpoints. *

 

In relation to the complexity of the primary endpoints, we do appreciate this point and assure the reviewer that we thought very carefully about this and had several discussions with the several independent review boards that have approved this protocol.  We felt that maintaining the hypothesis-driven study design with the analysis of the 4 primary study endpoints is appropriate taking into consideration the stage of development of IHAT and the fact that at the end of this trial we must be able to make a ‘go/no-go’ decision to move to the next stages of trials in resource-poor settings. The ‘go’ decision to continue development of IHAT in these settings will only be justified if we determine in this trial if IHAT can help correct iron deficiency anaemia without promoting diarrhoea, which is the largest limitation of  current supplements. This is why we maintained the current 4 primary endpoints. 

- *Is this a Phase 2a? efficacy trial to test non-inferiority of IHAT to treat anaemia compared to FeSo4 and safety of IHAT in this population.*

This is a Phase II where we test both efficacy and safety as primary endpoint. We have added this information to the abstract.

 

- Secondary endpoints could be referred to mechanistic endpoints. Further, mechanistic assessments, to test whether IHAT reduces the burden of enteric pathogens, morbidity, and intestinal inflammation, and that it does not cause detrimental changes to the gut microbiome, particularly in relation to *Lactobacillaceae*, *Bifidobacteriaceae *and *Enterobacteriaceae*.

 

Thank you for this suggestion, we have included this.

**-** *Many readers will not know how iron is absorbed in the body so the authors are encouraged to simplify and explain the basic physiology of iron absorption, the problem of oral iron compounds and therefore the novel properties of IHAT.*

The protocol is already quite long and we don’t have much room to explain iron absorption and metabolism. We do highlight better in the introduction what is unique about IHAT in relation to how current oral iron is absorbed. So this should help the reader to understand the novelty.

**- *Introduction***

*The authors provide an overview of ID and Anaemia. However, data on YLD and DALY are from adults not specific to children. They then talk about data in children. Please add a sentence on how ID and anaemia impact childhood development and illness to bring these two points closer together.*

*Also perhaps explain the difference between ID and Anaemia or simply refer to the problem as IDA throughout*

Thank you, we have now done this in the revised version.

 

- *Adding more sub-headings for clarity.*

 

This has been done, thank you.  

 

- ***Protocol*** *Remove comparator from this paragraph as that is a method.*

 

We moved this paragraph to the design.

***- Criteria***

*Why exclude previous / current iron supplements – as if still have IDA then this is failure of therapy.*

They are excluded because treatment failure is a secondary outcome of the trial and we did not want to have children currently taking iron supplements acting as confounders. But in practice, we did not have to exclude any child due to them taking iron supplements currently since none of the ~ 1500 children we screened had been screened for anaemia or was taking iron supplements in the last year.

*- Is haemoglobinopathy excluded?*

Yes we had included these in the ‘chronic conditions’ criteria but now we have separated them.

 

- *Childhood blood testing can be difficult – was this a criteria for inclusion i.e. a child must be well and able to give a venous blood sample?*

 

Yes, these criteria are part of our inclusion (ability to follow the protocol which includes the blood sampling). Our field nurses are very experienced and we have less than 5% of children where we have been unable to collect venous blood in one of the study visits.

*- Conducting, clinical trials in this setting is very difficult. There is clearly a labour intensive methodology. Has this been validated (i.e. are these standardised methods in this setting) please provide a reference.*

Agreed! There were no existing validated procedures for our field procedures or for many of the data and lab procedures. We spend the 1 year implementation period of the trial designing new standardised protocols for all trial procedures (lab, field and data) and have currently 25 standardised study specific procedures that we are using in IHAT-GUT and that we can make available to other researchers who would like to adapt them to their own studies. 

*-  The trial preparations have been manufactured to enable concealment and therefore blinding of the children. Are there blinded and unblinded assessors?*

*How is concealment bias ensured if there are weekly hb testing - are these results kept blinded also do all children attend or only some – will there be reporting bias as only the sicker children return for repeat testing?*

The trial is double-blinded. All participants and field and lab staff are blinded. Each child has a completely unique ‘drug’ code and therefore we don’t even know which children are in the same treatment arm. Haemoglobin in 3 months does not change that much and the field staff who measure Hb every week cannot determine which children will be on iron or placebo.

All children return to the clinics weekly unless they are travelling or mothers busy (~15% maximum of missed visits) and this does not depend on their well status.

Children do additional visits to the clinics if they have an adverse event.

*- Will children and healthcare workers be asked if they have an idea of what intervention was given to ensure lack of bias? It may be clear that a child’s health is improving and this may in turn lead to higher compliance compared to a group where the intervention is causing side effects.*

The health of every child in the study is improving simply due to all the extra care they receive whilst taking part in the study, with daily visits by the field staff and weekly visits to a nurse. All children that are sick with fever, diarrhoea etc are immediately treated. We must remember, this is a setting where there is very limited health care and children hardly ever would see a nurse or clinician unless they were severely sick. This is why it was so crucial in this study to have the placebo arm to determine the true effects of giving iron above just providing standard health care an no iron.

**- *Outcomes***

*Resolving iron deficiency and anaemia correction are two separate endpoints. What is the definition of resolving iron deficiency and also for correcting anaemia. A patient can have no anaemia but be iron deficient and vice versa.*

The model we will use for iron deficiency in the presence of inflammation is referenced and we will use the recommended method of the BRINDA group.

*- It seems the endpoint is Hb correction but hb rise > 1g/dl is not the same as an absolute correction of anaemia. Should this endpoint be change in Hb?*

This is justified in the Discussion.

 

- *If 705 to be consented and enrolled please use this number throughout for enrolment to the trial.*

 

We have changed this.

***- Discussion***

*As many of the patients have been enrolled, can the authors please highlight any interim analysis and also (I assume) the DSMC have approved continuation.*

Thanks, we have added a statement on this.
